# Supplementary material for: Pyruvate Dehydrogenase Kinase 1 inhibition mediated oxidative phosphorylation enhancement in cartilage promotes osteoarthritis progression
Source: BMC Musculoskelet Disord. 2023 Jul 20;24:597. doi: 10.1186/s12891-023-06585-6 (PMC10357736; doi:10.1186/s12891-023-06585-6)
Supplement: Supplementary file 1 — Additional file 1. [file 12891_2023_6585_MOESM1_ESM.zip › MF.Gsea.1653627316242/GOMF_NEUTRAL_AMINO_ACID_TRANSMEMBRANE_TRANSPORTER_ACTIVITY.html]

Details for gene set GOMF\_NEUTRAL\_AMINO\_ACID\_TRANSMEMBRANE\_TRANSPORTER\_ACTIVITY[GSEA]

|  || Dataset | 55457\_collapsed\_to\_symbols.cls.cls #OA\_versus\_normal.cls.cls #OA\_versus\_normal\_repos |
| Phenotype | cls.cls#OA\_versus\_normal\_repos |
| Upregulated in class | normal |
| GeneSet | GOMF\_NEUTRAL\_AMINO\_ACID\_TRANSMEMBRANE\_TRANSPORTER\_ACTIVITY |
| Enrichment Score (ES) | -0.5132504 |
| Normalized Enrichment Score (NES) | -1.7525887 |
| Nominal p-value | 0.0033955858 |
| FDR q-value | 0.08149887 |
| FWER p-Value | 0.74 |
Table: GSEA Results Summary

  

Fig 1: Enrichment plot: GOMF\_NEUTRAL\_AMINO\_ACID\_TRANSMEMBRANE\_TRANSPORTER\_ACTIVITY      
 Profile of the Running ES Score & Positions of GeneSet Members on the Rank Ordered List

  

| SYMBOL | TITLE | RANK IN GENE LIST | RANK METRIC SCORE | RUNNING ES | CORE ENRICHMENT || 1 | SLC1A4 | solute carrier family 1 member 4 [Source:HGNC Symbol;Acc:HGNC:10942] | 1372 | 0.447 | -0.0640 | No |
| 2 | SLC38A6 | solute carrier family 38 member 6 [Source:HGNC Symbol;Acc:HGNC:19863] | 3060 | 0.244 | -0.1718 | No |
| 3 | SFXN3 | sideroflexin 3 [Source:HGNC Symbol;Acc:HGNC:16087] | 3087 | 0.241 | -0.1508 | No |
| 4 | SLC43A1 | solute carrier family 43 member 1 [Source:HGNC Symbol;Acc:HGNC:9225] | 3384 | 0.213 | -0.1535 | No |
| 5 | SLC6A14 | solute carrier family 6 member 14 [Source:HGNC Symbol;Acc:HGNC:11047] | 4634 | 0.101 | -0.2409 | No |
| 6 | SLC38A7 | solute carrier family 38 member 7 [Source:HGNC Symbol;Acc:HGNC:25582] | 5916 | 0.001 | -0.3403 | No |
| 7 | SLC7A11 | solute carrier family 7 member 11 [Source:HGNC Symbol;Acc:HGNC:11059] | 6470 | -0.040 | -0.3795 | No |
| 8 | SLC1A2 | solute carrier family 1 member 2 [Source:HGNC Symbol;Acc:HGNC:10940] | 7026 | -0.082 | -0.4148 | No |
| 9 | SLC6A5 | solute carrier family 6 member 5 [Source:HGNC Symbol;Acc:HGNC:11051] | 7439 | -0.114 | -0.4360 | No |
| 10 | SLC36A1 | solute carrier family 36 member 1 [Source:HGNC Symbol;Acc:HGNC:18761] | 7670 | -0.132 | -0.4412 | No |
| 11 | SLC1A1 | solute carrier family 1 member 1 [Source:HGNC Symbol;Acc:HGNC:10939] | 8157 | -0.167 | -0.4631 | No |
| 12 | SLC7A10 | solute carrier family 7 member 10 [Source:HGNC Symbol;Acc:HGNC:11058] | 8492 | -0.193 | -0.4706 | No |
| 13 | SLC1A7 | solute carrier family 1 member 7 [Source:HGNC Symbol;Acc:HGNC:10945] | 8504 | -0.194 | -0.4529 | No |
| 14 | SLC6A9 | solute carrier family 6 member 9 [Source:HGNC Symbol;Acc:HGNC:11056] | 8704 | -0.209 | -0.4484 | No |
| 15 | SLC7A9 | solute carrier family 7 member 9 [Source:HGNC Symbol;Acc:HGNC:11067] | 9539 | -0.277 | -0.4868 | Yes |
| 16 | SLC1A3 | solute carrier family 1 member 3 [Source:HGNC Symbol;Acc:HGNC:10941] | 9656 | -0.288 | -0.4684 | Yes |
| 17 | SLC25A38 | solute carrier family 25 member 38 [Source:HGNC Symbol;Acc:HGNC:26054] | 9904 | -0.312 | -0.4578 | Yes |
| 18 | SLC6A7 | solute carrier family 6 member 7 [Source:HGNC Symbol;Acc:HGNC:11054] | 10185 | -0.339 | -0.4472 | Yes |
| 19 | SLC7A8 | solute carrier family 7 member 8 [Source:HGNC Symbol;Acc:HGNC:11066] | 10228 | -0.344 | -0.4177 | Yes |
| 20 | SERINC5 | serine incorporator 5 [Source:HGNC Symbol;Acc:HGNC:18825] | 10352 | -0.356 | -0.3933 | Yes |
| 21 | SLC38A3 | solute carrier family 38 member 3 [Source:HGNC Symbol;Acc:HGNC:18044] | 10443 | -0.366 | -0.3654 | Yes |
| 22 | SLC38A1 | solute carrier family 38 member 1 [Source:HGNC Symbol;Acc:HGNC:13447] | 10617 | -0.385 | -0.3421 | Yes |
| 23 | SLC1A6 | solute carrier family 1 member 6 [Source:HGNC Symbol;Acc:HGNC:10944] | 10626 | -0.386 | -0.3060 | Yes |
| 24 | SFXN1 | sideroflexin 1 [Source:HGNC Symbol;Acc:HGNC:16085] | 10880 | -0.414 | -0.2862 | Yes |
| 25 | MFSD12 | major facilitator superfamily domain containing 12 [Source:HGNC Symbol;Acc:HGNC:28299] | 10965 | -0.426 | -0.2521 | Yes |
| 26 | SLC6A20 | solute carrier family 6 member 20 [Source:HGNC Symbol;Acc:HGNC:30927] | 10982 | -0.428 | -0.2126 | Yes |
| 27 | SERINC3 | serine incorporator 3 [Source:HGNC Symbol;Acc:HGNC:11699] | 11087 | -0.442 | -0.1785 | Yes |
| 28 | SLC38A2 | solute carrier family 38 member 2 [Source:HGNC Symbol;Acc:HGNC:13448] | 11722 | -0.536 | -0.1766 | Yes |
| 29 | SLC6A6 | solute carrier family 6 member 6 [Source:HGNC Symbol;Acc:HGNC:11052] | 12128 | -0.626 | -0.1484 | Yes |
| 30 | SLC7A5 | solute carrier family 7 member 5 [Source:HGNC Symbol;Acc:HGNC:11063] | 12203 | -0.646 | -0.0926 | Yes |
| 31 | SLC1A5 | solute carrier family 1 member 5 [Source:HGNC Symbol;Acc:HGNC:10943] | 12501 | -0.748 | -0.0443 | Yes |
| 32 | SLC3A2 | solute carrier family 3 member 2 [Source:HGNC Symbol;Acc:HGNC:11026] | 12587 | -0.789 | 0.0243 | Yes |
Table: GSEA details [plain text format]

  

Fig 2: GOMF\_NEUTRAL\_AMINO\_ACID\_TRANSMEMBRANE\_TRANSPORTER\_ACTIVITY      
 Blue-Pink O' Gram in the Space of the Analyzed GeneSet

  

Fig 3: GOMF\_NEUTRAL\_AMINO\_ACID\_TRANSMEMBRANE\_TRANSPORTER\_ACTIVITY: Random ES distribution      
 Gene set null distribution of ES for **GOMF\_NEUTRAL\_AMINO\_ACID\_TRANSMEMBRANE\_TRANSPORTER\_ACTIVITY**
